# The Effects of a High-Protein Diet on Bone Mineral Density in Exercise-Trained Women: A 1-Year Investigation

**DOI:** 10.3390/jfmk3040062

**Published:** 2018-12-05

**Authors:** Jose Antonio, Anya Ellerbroek, Cassandra Carson

**Affiliations:** Department of Health and Human Performance, Nova Southeastern University, Davie, FL 32004; USA

**Keywords:** bone, DXA, diet, protein, exercise

## Abstract

The effects of long-term high-protein consumption (i.e., >2.2 g/kg/day) are unclear as it relates to bone mineral content. Thus, the primary endpoint of this investigation was to determine if consuming a high-protein diet for one year affected various parameters of body composition in exercise-trained women. This investigation is a follow-up to a prior 6-month study. Subjects were instructed to consume a high-protein diet (>2.2 g/kg/day) for one year. Body composition was assessed via dual-energy X-ray absorptiometry (DXA). Subjects were instructed to keep a food diary (i.e., log their food ~three days per week for a year) via the mobile app MyFitnessPal^®^. Furthermore, a subset of subjects had their blood analyzed (i.e., basic metabolic panel). Subjects consumed a high-protein diet for one year (mean ± SD: 2.3 ± 1.1 grams per kilogram body weight daily [g/kg/day]). There were no significant changes for any measure of body composition over the course of the year (i.e., body weight, fat mass, lean body mass, percent fat, whole body bone mineral content, whole body T-score, whole body bone mineral density, lumbar bone mineral content, lumbar bone mineral density and lumbar T-score). In addition, we found no adverse effects on kidney function. Based on this 1-year within-subjects investigation, it is evident that a diet high in protein has no adverse effects on bone mineral density or kidney function.

## 1. Introduction

It has been postulated that high protein consumption (i.e., greater than the recommended daily allowance [RDA]) may pose a health risk in certain conditions [1,2,3,4]; however, these concerns are not applicable to a healthy exercise-trained population. Protein restriction might play a key role in the management of chronic kidney disease (CKD) [3]. However, according to Levey et al., a 2- to 3-year intervention of dietary protein restriction had no conclusive effects on renal health in those with kidney disease [1]. In a recent systematic review, it was found that a low-protein diet showed no benefit regarding renal function in patients with either type 1 or 2 diabetic nephropathy [4]. Furthermore, even if one assumes that dietary protein restriction is indeed helpful for CKD patients, it is erroneous to conflate health issues in those with renal dysfunction to exercise-trained men and women.

It is known that the kidneys play a vital role in bone development and metabolism. In fact, the kidneys produce several factors that regulate different stages of bone development and repair (e.g., 1,25(OH)2D3, Klotho, bone morphogenetic protein-7, and erythropoietin) [5]. Thus, there is a notion that renal dysfunction due to excessive protein intake might play a role in bone health [6]. There is acute data that shows that after exhaustive running, immediate ingestion of carbohydrate and protein decreases bone resorption marker concentrations (i.e., C-terminal telopeptide of type I collagen [beta-CTX]) and increases bone formation marker concentrations (i.e., N-terminal propeptides of type 1 procollagen [P1NP]) [7]. This would suggest a positive effect on bone mineral content. Our prior work showed that consuming a high-protein diet for one year has no negative effect on kidney or liver function in male bodybuilders [8]. Whether bone health is compromised is based on the acid-ash hypothesis. The acid-ash diet hypothesis suggests that acid from a typical meat-containing Western diet may cause bone demineralization with the consequent excretion of calcium, thus leading to the loss of bone mineral content [9]. It is further known that exercise plays a crucial role in maintaining bone mineral content and density [10,11]. Therefore, the effects of a combination of chronic exercise training coupled with a high-protein diet on parameters of bone health are not entirely known. Our lab conducted a 6-month investigation, which demonstrated that a high-protein diet (2.8 g/kg/day) had no adverse effects on bone mineral in exercise-trained women [12]. Thus, the purpose of this follow-up investigation was to determine if consuming a high-protein diet for 12 months affected body composition (i.e., bone mineral content, bone mineral density, lean body mass, fat mass, etc.) as well as measures of metabolic health (i.e., renal function).

## 2. Materials and Methods 

### 2.1. Participants

Twenty-seven exercise-trained female subjects (mean ± SD: age 37 ± 9 years; height 167 ± 7 cm; weight 59.3 ± 5.0 kg) completed this single-arm trial. This study was in part an extension of previously published work from our lab [12]. In the current investigation, subjects had to be performing a minimum of resistance- and/or aerobic-training sessions three times per week for at least the last year to qualify as “exercise-trained.” The research subjects’ mean training time per week of aerobic and resistance training was 4.4 and 3.1 h/week, respectively. Subjects came to the laboratory on three occasions for testing (baseline, 6 months and 1 year). In accordance with the Helsinki Declaration, the university’s Institutional Review Board approved all human subjects procedures. Written informed consent was obtained prior to participation.

### 2.2. Body Composition

Subjects had their height and weight determined using a calibrated scale. Body composition and bone mineral density were assessed with a dual-energy X-ray absorptiometry machine (DXA) (Model: Hologic Horizon W; Hologic Inc., Danbury, CT, USA) [13]. Subjects were instructed to come to the laboratory after at least a 3-h fast and no prior exercise that day. All testing was performed between 11:00 a.m. and 4:00 p.m. Subjects typically came to the lab at the same time for each of the three testing dates. Quality control calibration procedures were performed on a spine phantom. Subjects wore typical athletic clothing and removed all metal jewelry. They were positioned supine on the DXA within the borders delineated by the scanning table. Each whole-body scan took approximately seven minutes. In addition, a lumbar spine scan was performed. Subjects were positioned supine on the scanning table with their legs resting on a pad that allowed them to have their knee and hip joint at approximately a 90-degree angle. The lumbar scan took approximately 15 s. The lumbar spine is considered the most appropriate site for monitoring the effect of a treatment [13].

### 2.3. Metabolic Panel

A smaller subset of subjects agreed to have their blood analyzed for markers of renal function. They went to a Quest Diagnostics facility to have their blood drawn in a fasted state. Quest Diagnostics is a laboratory testing service that offers a variety of laboratory tests. Thus, for a metabolic panel, the following was assessed: blood urea nitrogen, creatinine, glucose, albumin, CO_2_, calcium, sodium, potassium and chloride.

### 2.4. Diet and Exercise

Subjects were instructed to consume a high-protein (>2.2 g/kg/day) diet during the treatment period. Our pool of subjects typically logged their food on a regular basis. Thus, they were well versed at tracking their intake. Subjects had the choice of consuming protein via their typical diet (i.e., eat more protein-containing foods) or the consumption of protein powder. However, subjects were not required to consume protein powder. Dymatize^®^ provided whey and casein protein powder in the event that subjects needed a convenient way to consume more protein. Subjects were also instructed to keep a food log (three times per week) on the MyFitnessPal mobile app [14]. Thus, a total of ~150 daily food logs were collected during the year. All subjects had prior experience using the mobile app and as such were quite skilled at logging their food intake. The subjects’ training regimen was not altered in any way; thus, each subject self-selected their exercise regimen.

### 2.5. Statistical Analysis

A repeated measures analysis of variance was used to assess differences in body composition at baseline, 6 months and 1 year. A paired *t* test was used to analyze blood data from the basic metabolic panel. All data is presented as the mean ± SD. GraphPad (Prism 6, San Diego, CA, USA) software was used for statistical analyses.

## 3. Results

### 3.1. Energy and Macronutrient Intake

Twenty-seven exercise-trained women completed this investigation (mean ± SD. age: 37 ± 9 years; height: 167 ± 7 cm; body weight: 59.3 ± 5.0 kg). Over the course of the year, they consumed a high-protein diet (2.3 ± 1.1 g/kg/day) (Table 1).

### 3.2. Body Composition

**Figure 1:**
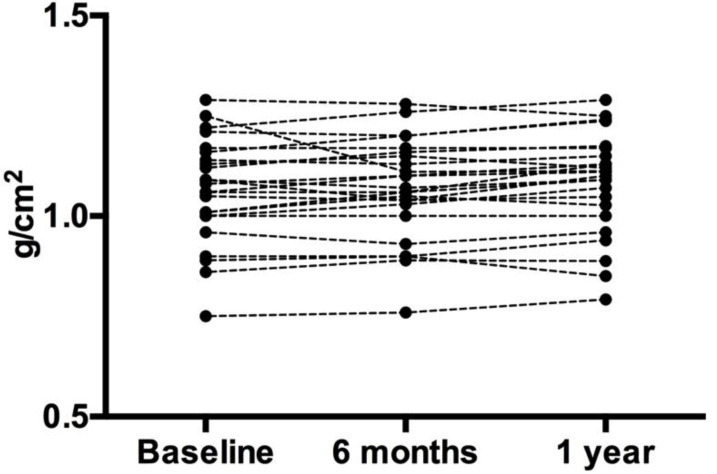
Lumbar Bone Mineral Density. Each circle represents an individual data point. Despite the slight variation in individual data, the overall group mean did not change.

**Figure 2:**
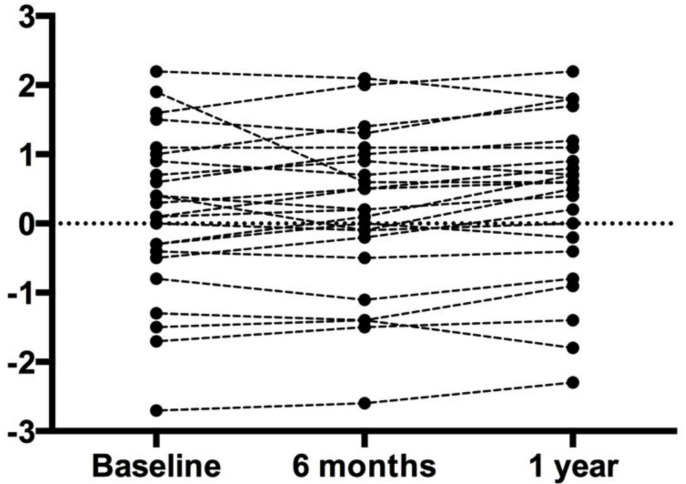
Lumbar T-Score. Each circle represents an individual data point. Despite the slight variation in individual data, the overall group mean did not change.

There were no changes over the course of the treatment period for any body composition measure (i.e., body weight, fat mass, lean body mass, percent body fat, bone mineral content, bone mineral density, total body T-score, Lumbar bone mineral density, lumbar bone mineral content, lumbar T-score) (Table 2 and Figure 1 and Figure 2).

### 3.3. Basic Metabolic Panel

There were no adverse effects in any measure of health as determined by a basic metabolic panel (i.e., glucose, blood urea nitrogen (BUN), creatine, estimated glomerular filtration rate, BUN/creatinine ratio, sodium, potassium, chloride, CO_2_, and calcium) (Table 3).

## 4. Discussion

This is the first 1-year investigation that has examined the effects of a chronic high-protein diet on measures of bone mineral content or density in exercise-trained women. Subjects consumed 2.3 g/kg/day over a 1-year period; nonetheless, there were no changes in whole-body or lumbar bone mineral density, bone mineral content or T-score.

There have been other investigations that have made direct measures of bone health after the consumption of a higher protein diet. Our prior work showed that a high-protein diet (2.8 g/kg/day versus 1.5 g/kg/day) has no effect on bone mineral content, bone mineral density or T-scores [12]. Also, a 1-year treatment that consisted of dietary soy protein and/or soy isoflavones on bone health in late postmenopausal women found that neither soy protein nor isoflavones (in combination or alone) had any effect on BMD. However, the protein intake from that study was actually quite low (~0.9 g/kg/day) [15]. Ballard et al. performed a 6-month investigation on protein supplementation and bone health [16]. They discovered that additional protein over the 6-month treatment duration had no effect on BMD or bone size in young adults (18–25 years). The protein intake of the subjects in the Ballard et al. investigation was also fairly low (1.0–1.2 g/kg/day). In addition, whey protein supplementation (total daily protein intake of 1.0–1.1 g/kg/day) in older men and women (>60 years) had no effect on bone mass. On the other hand, it did increase truncal lean mass after an 18-month treatment period [17].

Protein supplementation studies in non-athletic or sedentary populations tend to use fairly low doses of protein intake. It would be erroneous to apply those studies to exercise-trained individuals. The data clearly show that individuals that exercise regularly are advised to consume at least twice the recommended daily allowance (RDA) of protein [18,19,20,21,22]. The protein intake in the current investigation averaged 2.3 g/kg/day over the 1-year treatment, which is clearly a “high” intake of dietary protein. Other studies have used much lower doses of dietary protein [15,16,17]. If consuming a high-protein diet induced harm, one would reasonably expect to see this after one year of consuming 2.3 g/kg/day. Yet we found no effect on any measure of bone mineral content or density.

This is the second investigation that has used exercise-trained women in ascertaining the effects of a high-protein diet. This is relevant because exercise-trained individuals purposefully consume a higher protein diet [8,18,19,21,23,24,25,26,27,28,29]. Conversely, sedentary individuals do not typically consume a high-protein diet. One of the main limitations of this investigation is the use of self-reported dietary intakes. For instance, the total energy intake of our subjects seemed rather low for active individuals (28.7 kcal/kg/day). These individuals should be consuming greater than 2000 calories per day [30]. Therefore, it is possible that our subjects were underreporting total energy intake, particularly from carbohydrate and fat. However, that would also suggest that they were underreporting dietary protein. In spite of the drawbacks in using dietary records, it is evident that high protein consumption has no harmful effect on bone mineral content or density.

In addition to the lack of harm on bone mineral density, we also discovered that a chronic high-protein diet in trained women had no effect on renal function. This is in agreement with prior work on exercise-trained individuals that consumed a high intake of protein [8].

## 5. Conclusions

In conclusion, exercise-trained female subjects that consume a diet that is approximately three times greater than the RDA for protein experience no harmful effects on bone mineral density or content. Nor were there any harmful effects on renal function. Thus, it is evident that exercise-trained women can consume a chronically high-protein diet with no adverse effects.

## Figures and Tables

**Table 1:** Dietary Intake.

Diet	Mean Intake over the Entire Year
Energy (kcal)	1708 ± 398
Protein (g)	139 ± 59
Carbohydrate (g)	158 ± 52
Fat (g)	57 ± 12
Cholesterol (mg)	397 ± 365
Sodium (mg)	2036 ± 896
Sugar (g)	52 ± 24
Fiber (g)	24 ± 13
Energy (kcal/kg/d)	28.7 ± 8.0
Protein (g/kg/d)	2.3 ± 1.1
Carbohydrate (g/kg/d)	2.7 ± 0.9
Fat (g/kg/d)	1.0 ± 0.2

Data are expressed as the mean ± SD. *n* = 27. Legend: d—day; g—gram; kcal—kilocalorie; kg—kilogram; mg—milligram.

**Table 2:** Body Composition.

	Baseline	6 Month	12 Month
Body Weight (kg)	59.3 ± 5.0	59.5 ± 5.0	59.6 ± 5.8
Fat Mass (kg)	14.7 ± 2.6	14.5 ± 3.5	14.7 ± 3.4
Lean Body Mass (kg)	42.1 ± 4.3	42.6 ± 4.3	42.4 ± 4.6
% Body Fat	24.8 ± 4.0	24.3 ± 5.3	24.6 ± 4.8
Bone Mineral Content (kg)	2.4 ± 0.3	2.4 ± 0.3	2.4 ± 0.3
Bone Mineral Density (g/cm^2^)	1.22 ± 0.09	1.21 ± 0.10	1.21 ± 0.09
Total Body T-Score	1.4 ± 1.1	1.3 ± 1.2	1.2 ± 1.1
Lumbar Bone Mineral Content (g)	67.2 ± 10.6	67.9 ± 10.1	69.5 ± 9.00
Lumbar Bone Mineral Density (g/cm^2^)	1.07 ± 0.13	1.07 ± 0.12	1.09 ± 0.12
Lumbar T-Score	0.23 ± 1.18	0.20 ± 1.10	0.35 ± 1.09

Data are expressed as the mean ± SD. *n* = 27. There were no significant differences between groups. Legend: cm—centimeter; g—grams; kg—kilogram. The T-score is the number of standard deviations that one’s bone mineral density is above or below the average. Scale for T-score: −1 and above is normal. Between −1 and −2.5 is osteopenia. −2.5 or below is osteoporosis.

**Table 3:** Basic metabolic panel.

	Baseline	1 Year	Reference Range
Glucose mg/dL	90 ± 11	95 ± 6	65–99
BUN mg/dL	19 ± 5	19 ± 4	7–25
Creatinine mg/dL	0.89 ± 0.14	0.85 ± 0.07	0.60–1.35
eGFR	85 ± 13	89 ± 11	≥ 60 mL/min/1.73 m^2^
BUN/Creatinine ratio	22 ± 6	23 ± 4	6–22
Sodium mmol/L	139 ± 1	139 ± 2	135–146
Potassium mmol/L	4.5 ± 0.3	4.2 ± 0.3	3.5–5.3
Chloride mmol/L	105 ± 2	105 ± 2	98–110
CO_2_ mmol/L	27 ± 3	27 ± 2	19–30
Calcium mg/dL	9.2 ± 0.3	9.2 ± 0.3	8.6–10.3

Data are mean ± SD. Only a small group of subjects (*n* = 7) agreed to having their blood analyzed. There were no differences between baseline and 1 year. Legend: BUN—blood urea nitrogen; dL—deciliter; eGFR—estimated glomerular filtration rate; L—liter; mg—milligram; mmol—millimole.
